# *Nigella sativa* for the treatment of COVID‐19 patients: A rapid systematic review and meta‐analysis of randomized controlled trials

**DOI:** 10.1002/fsn3.3906

**Published:** 2023-12-27

**Authors:** Mohammad Umer, Aiman Naveed, Qanita Maryam, Huzaifa Ahmad Cheema, Abia Shahid, Alaa Hamza Hermis, Sampath Chinnam, Sarya Swed, Syeda Sahra

**Affiliations:** ^1^ Division of Infectious Diseases, Department of Medicine King Edward Medical University Lahore Pakistan; ^2^ Sir Ganga Ram Hospital Lahore Pakistan; ^3^ Nursing College Al‐Mustaqbal University Hillah Babylon Iraq; ^4^ Department of Chemistry M. S. Ramaiah Institute of Technology (Affiliated with Visvesvaraya Technological University, Belgaum) Bengaluru Karnataka India; ^5^ Faculty of Medicine Aleppo University Aleppo Syria; ^6^ Department of Infectious Diseases University of Oklahoma Health Sciences Center Oklahoma City Oklahoma USA

**Keywords:** COVID‐19, cumin, meta‐analysis, *Nigella sativa*, SARS‐CoV‐2

## Abstract

*Nigella sativa* is an herbal therapy for various afflictions. It has some potential to be a promising option as an efficacious treatment for COVID‐19 patients that can contribute to global healthcare as a relatively cheap therapy but evidence of its use from randomized controlled trials (RCTs) is limited. Therefore, to explore the effect of *N. sativa* in combating COVID‐19, we undertook this meta‐analysis. We searched several databases to retrieve all RCTs investigating *N. sativa* for the treatment of COVID‐19 as compared to placebo or standard care. We used RevMan 5.4 for all analyses with risk ratio (RR) or odds ratio (OR) as the effect measures. We included a total of seven RCTs in this review. *N. sativa* significantly reduced the risk of all‐cause mortality in patients with COVID‐19 compared to the control group (RR 0.27, 95% CI: 0.10 to 0.72; *I* ^2^ = 0%). *N. sativa* significantly reduced the rate of viral PCR positivity (RR 0.62, 95% CI: 0.39 to 0.97; *I* ^2^ = 0%). We did not find any significant difference in the risk of hospitalization (RR 0.26, 95% CI: 0.04 to 1.54; *I* ^2^ = 0%) and the rate of no recovery (OR 0.48, 95% CI: 0.20 to 1.15; *I* ^2^ = 84%) between the two groups. *N. sativa* is an easily available herbal medicine that may decrease the risk of mortality and improve virological clearance in COVID‐19 patients. However, our results are limited by the small number of RCTs available. Further large‐scale RCTs are needed to better understand the anti‐inflammatory and antiviral effects of *N. sativa* in COVID‐19 patients.

## Graphical abstract

*N. sativa* is an easily available herbal medicine that may decrease the risk of mortality and improve virological clearance in COVID‐19 patients. However, our results are limited by the small number of RCTs available. Further large‐scale RCTs are needed to better understand the effects of *N. sativa* as an antiviral and anti‐inflammatory agent in COVID‐19 patients.

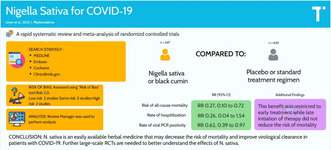

## 1 INTRODUCTION

The coronavirus disease (COVID‐19) since evolving into a pandemic has become and continues to be a global health crisis, significantly contributing to mortality and morbidity rates worldwide. The pathogenesis of this disease is incompletely understood. Data suggest that the initiating event is the irregular activation of the immune system by the virus which results in elaboration of a cytokine storm as well as recruitment of macrophages and neutrophils. This proinflammatory state promotes activation of the complement system and neutrophil extracellular traps (NETs) which contribute to the activation of the coagulation cascade and bring about the subsequent thrombo‐inflammatory state (Borczuk & Yantiss, 2022). This manifests as tissue damage, organ failure, hypoxia, and eventual death. The search for effective treatments has been relentless, with researchers exploring various potential therapeutic options, including monoclonal antibodies, immunomodulators, antiviral agents, and repurposed drugs (Agarwal et al., 2020; Fatima et al., 2022). In addition, various traditional medicinal plants, such as quercetin and curcumin, have also been investigated and have demonstrated encouraging results (Cheema et al., 2023; Shafiee et al., 2023). *Nigella sativa*, also known as black seed or black cumin, has recently attracted attention for its potential therapeutic effects against COVID‐19 (Khazdair et al., 2021). *N. sativa* has long been used for its wide range of therapeutic properties in traditional settings. Its anti‐inflammatory, antioxidant, and antiviral effects as well as its influence on immunomodulation have given it this position in traditional medicine (Bordoni et al., 2019; Gholamnezhad et al., 2014). Several preliminary studies have strongly hinted that *N. sativa* may exert a positive influence on patients with COVID‐19, making it a promising candidate for further investigation and incorporation into treatment protocols (Koshak & Koshak, 2020).

According to a previous meta‐analysis, *N. sativa* has been found to reduce the risk of mortality in COVID‐19 patients; however, it did not evaluate other important clinical outcomes and did not include the most recent randomized controlled trials (RCTs) (Kow et al., 2023). In the current pandemic era, newer variants of COVID‐19 have been reported to be causing a milder disease and are associated with a low risk of mortality (Lorenzo‐Redondo et al., 2022). Therefore, interventions that improve other clinical outcomes, such as the risk of hospitalization and recovery rates are also of paramount importance. Hence, we conducted a meta‐analysis of RCTs to evaluate what effect *N. sativa* has on important outcomes in COVID‐19 patients.

## 2 METHODS

The Preferred Reporting Items for Systematic Review and Meta‐Analyses (PRISMA) guidelines (Page et al., 2021) were followed when reporting this preregistered study (PROSPERO CRD42023390278).

### 2.1 Search strategy and study selection

Using no filters or restrictions, a comprehensive literature search of MEDLINE (via PubMed), Embase, ProQuest Dissertations and Theses Global (PQDT), the Cochrane Library, and ClinicalTrials.gov, was conducted from the beginning to the 15th of May 2023. The following terms were used to construct a search strategy: “COVID‐19” “novel coronavirus”, “SARS‐CoV‐2”, “coronavirus disease”, “*Nigella sativa*”, and “black cumin”. Additional relevant articles were sought from the reference lists of relevant articles. The following criteria were used to determine whether a study should be included: (1) population: COVID‐19 patients of any hospitalization status or age; (2) intervention: *N. sativa* (irrespective of regimen); (3) Control: placebo or standard care; and (4) study design: RCTs. We excluded the studies that used *N. sativa* as a prophylactic agent.

### 2.2 Data extraction and quality assessment

Studies were selected after initial screening of titles and abstracts. This was followed by full‐text evaluations. The studies that met the inclusion criteria were short‐listed. Data were extracted into a data extraction sheet by two authors independently. Data regarding the design of the research, location of the trial, participants involved, intervention implemented, comparison group used, and outcomes were recorded. Quality of the included studies was ascertained using the revised Cochrane Risk of Bias Tool (RoB 2.0). Two reviewers independently reviewed each study to determine its risk of bias as “low”, “some concerns” or “high”. Discussion between the disagreeing reviewers was the method of choice for resolving disagreements followed by arbitration by a third reviewer if required.

### 2.3 Outcomes

The primary outcomes were the risk of all‐cause mortality and the rate of hospitalization. Our secondary outcomes were the proportion of patients with a positive viral PCR at follow‐up and the rate of no recovery (defined as the proportion of COVID‐19‐positive patients who had not recovered at follow‐up). For studies that reported recovery rates for multiple symptoms, we considered data for the worst symptom in our analysis.

### 2.4 Data analysis

We utilized RevMan version 5.4 for conducting all our analyses. To combine the data, we employed a random‐effects model with the DerSimonian and Laird variance estimator. The effect measures were reported as risk ratio (RR) or odds ratio (OR), with 95% confidence intervals. Heterogeneity was evaluated using both the Chi^2^ and *I* ^2^ statistics. To aid in interpreting the *I* ^2^ values, we referred to the Cochrane Handbook for Systematic Reviews of Intervention as the guide. Statistical significance for heterogeneity was set at *p* < .10 for the Chi^2^ test. For our primary outcomes, we performed subgroup analysis to assess any potential effect modification based on early initiation of therapy (within 5 days of symptom onset) compared to later initiation beyond that timeframe. Due to including fewer than 10 studies in our review, evaluating publication bias was not possible.

## 3 RESULTS

### 3.1 Study characteristics

**FIGURE 1:**
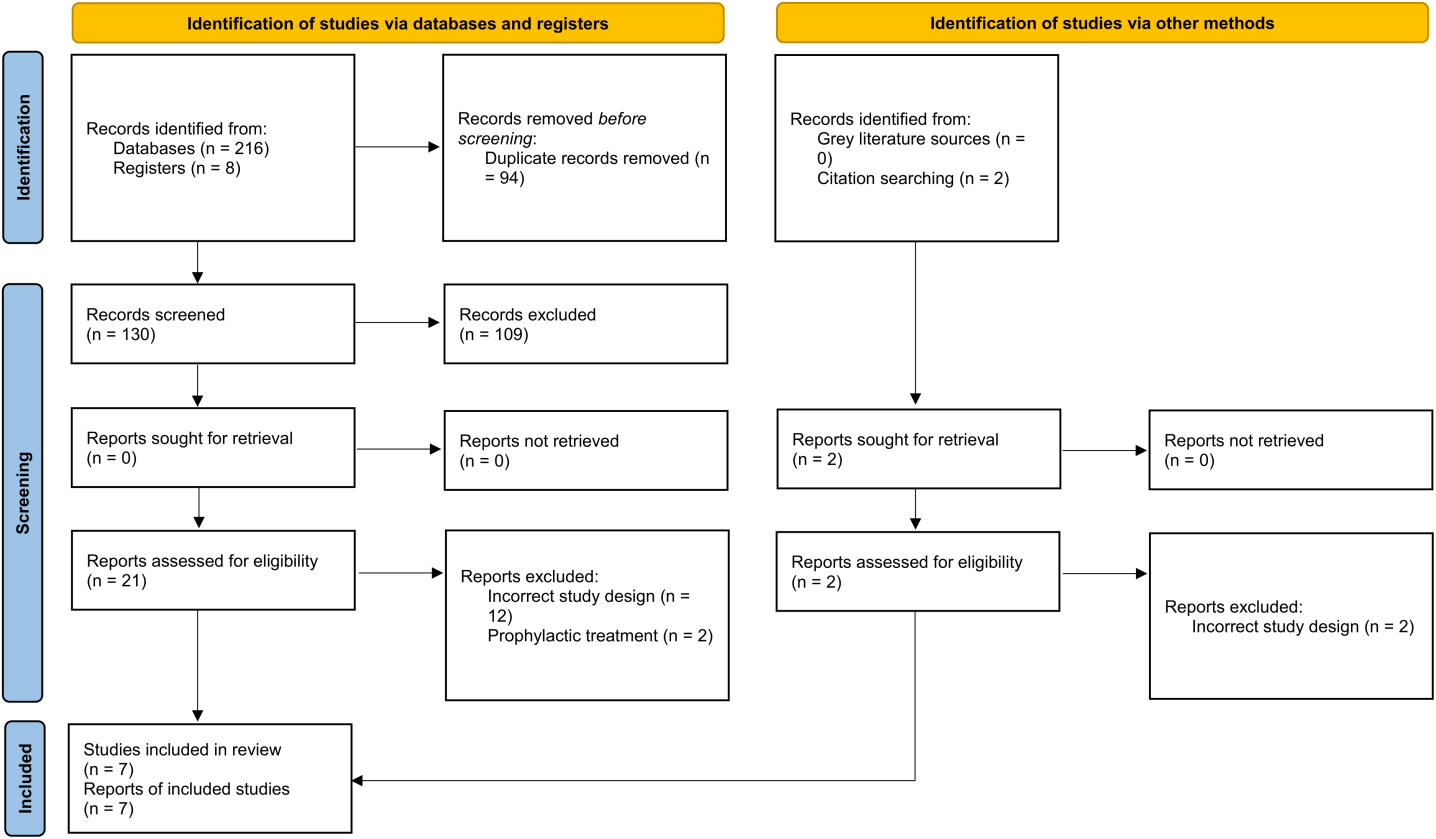
PRISMA 2020 flowchart.

A total of seven RCTs were included in the final review. The complete screening process is depicted in Figure 1. Of these, four were published in 2020, two in 2021, and one in 2022. All of the included studies were open‐label, except for two (Ashraf et al., 2022; Bencheqroun et al., 2022) which were double‐blinded. Three out of seven studies (Ali et al., 2021; Bencheqroun et al., 2022; Koshak et al., 2021) assessed the effects of *N. sativa* as a standalone therapy while the rest of the studies employed *N. sativa* in combination with other interventions. Two RCTs were classified as late treatment studies while the rest started *N. sativa* early. See Table 1 for details of all included studies.

**TABLE 1:** Detailed characteristics of included studies.

S. no	Study	Country	Study design	Age (median/mean)	Population	Regimen of *Nigella sativa* in the intervention group	Regimen of comparator intervention in the control group
1	Ashraf (2021)	Pakistan	Double blind	NA	313 outpatients, 157 treated with *Nigella sativa* and honey, 156 with standard care	Standard care along with 0.5 g/kg honey and 40 mg/kg NS BID (for 13 days)	Standard care: antipyretics, anticoagulants, antibiotics, steroids, supplemental oxygen, and mechanical ventilation
2	Haidiri (2020)	Iraq	Open label	NA	259 standard care 160 *Nigella* and standard care	40 mg/kg orally once daily for 14 days plus standard protocol	Standard care protocol
3	Koshak (2020)	Saudi Arabia	Open label	Mean ± SD: Total = 36 ± 11, Nigella = 35 ± 10, Control = 36 ± 12	Total of 183 participants 91 in *Nigella* Group 92 in control group	Standard care with oral NSO (500 mg twice daily postprandial for 10 days	Standard care
4	Bencheqroun (2022)	USA	Double blind	45.69 ± 17.35 Treatment: 45.48 ± 19.29 Control: 45.92 ± 15.27	Total of 55 participants, 29 received NS, 26 placebo	Standard therapy along with 500 mg capsules of NS, 3 capsules, twice a day for 14 days	Standard therapy
5	Said (2021)	Saudi Arabia	Open label	Median (Range): 41.5 (19–64) *Nigella sativa*: 29.0 (21.0–62.0) Standard: 26.0 (21.0–64.0)	30 participants in each group (Control, NS, Vitamin D, NS + Vitamin D)	Group 1: Total oral dose of 900 mg *Nigella sativa* in the form of 450 mg capsules, two capsules twice daily for 2 weeks and standard therapy Group 2: 2 tablets of 1000‐IU Vitamin D3 twice daily and standard therapy Group 3: *Nigella sativa* and Vitamin D3 in same dosing manner in addition to the standard therapy	Standard therapy: 500 mg Azithromycin, 1 capsule once daily for 5 days 1 g Vitamin C, 1 capsule once daily 50 mg Zinc, 1 capsule once daily 100 mg sachets of lactoferrin twice daily 10 mg Rivaroxaban tablet, once daily 500 mg Paracetamol
6	Setayesh (2020)	Iran	Open label	Mean: 59.1 ± 17.1 years	Total of 80, 40 in *Nigella* group, 40 in control	500 mg capsule of extract of the Glycyrrhiza *glabra, Punica granatum*, *Rheum palmatum*, and 500 mg capsule of *Nigella sativa* powder, TID for 7 days	Standard care protocol for Diagnosis and Treatment of Novel Coronavirus Pneumonia
7	Karimi (2020)	Iran	Open Label	49.755 ± 15. 370 Treatment: 48.72 ± 14.863 Control: 50.79 ± 15.878	358 hospitalized patients, 184 in herbal treatment group, and 174 in control	500 mg capsule containing herbal remedies and starch as filler, plus 500 mg capsule of NS, both capsules given BID, plus a decoction containing a sachet of powdered herbs, used TID (each time about 300 cc)	Standard care including: azithromycin, hydroxychloroquine, KALETRA® (lopinavir/ritonavir)

Abbreviations: BID, twice daily; IV, intravenous; NA, not available; PO, per os (by mouth); TID, thrice daily.

### 3.2 Risk‐of‐bias assessment

Two studies (Ashraf et al., 2022; Bencheqroun et al., 2022) had a low risk of bias while three studies showed some concerns due to missing outcome data (Koshak et al., 2021) or lack of information regarding allocation sequence concealment (Ali et al., 2021; Said et al., 2022). The remaining two studies (Karimi et al., 2021; Setayesh et al., 2022) had a high risk of bias (Figure S1).

### 3.3 Results of the meta‐analysis

#### 3.3.1 Primary outcomes

**FIGURE 2:**
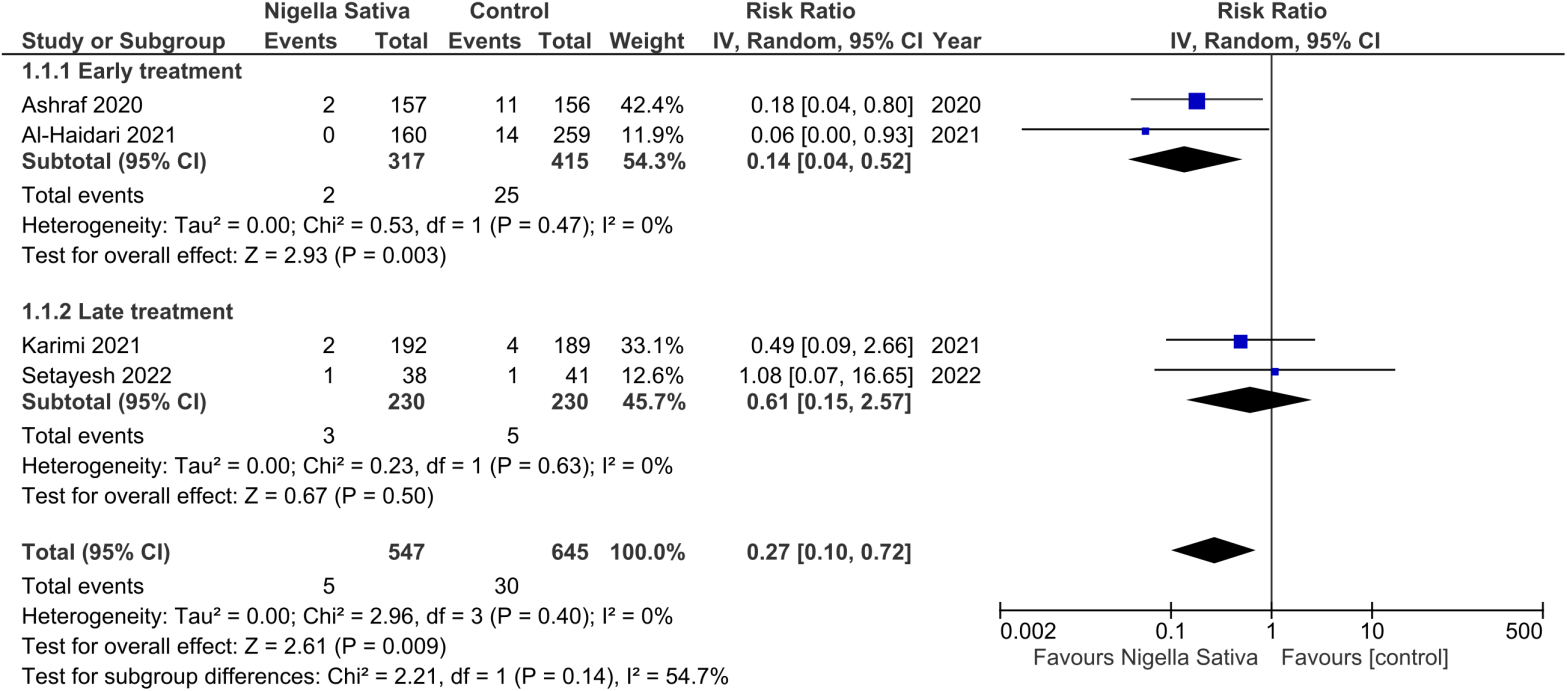
Effect of *Nigella sativa* on all‐cause mortality in COVID‐19 patients.

*N. sativa* significantly reduced the risk of all‐cause mortality in COVID‐19 patients compared to the control group (RR 0.27, 95% CI: 0.10 to 0.72; *I* ^2^ = 0%; Figure 2). This benefit was restricted to early treatment (RR 0.14, 95% CI: 0.04 to 0.52; *I* ^2^ = 0%) while late initiation of therapy did not reduce the risk of mortality (RR 0.61, 95% CI: 0.15 to 2.57; *I* ^2^ = 0%). However, the test for subgroup differences did not reach statistical significance (*p* = .14).

**FIGURE 3:**
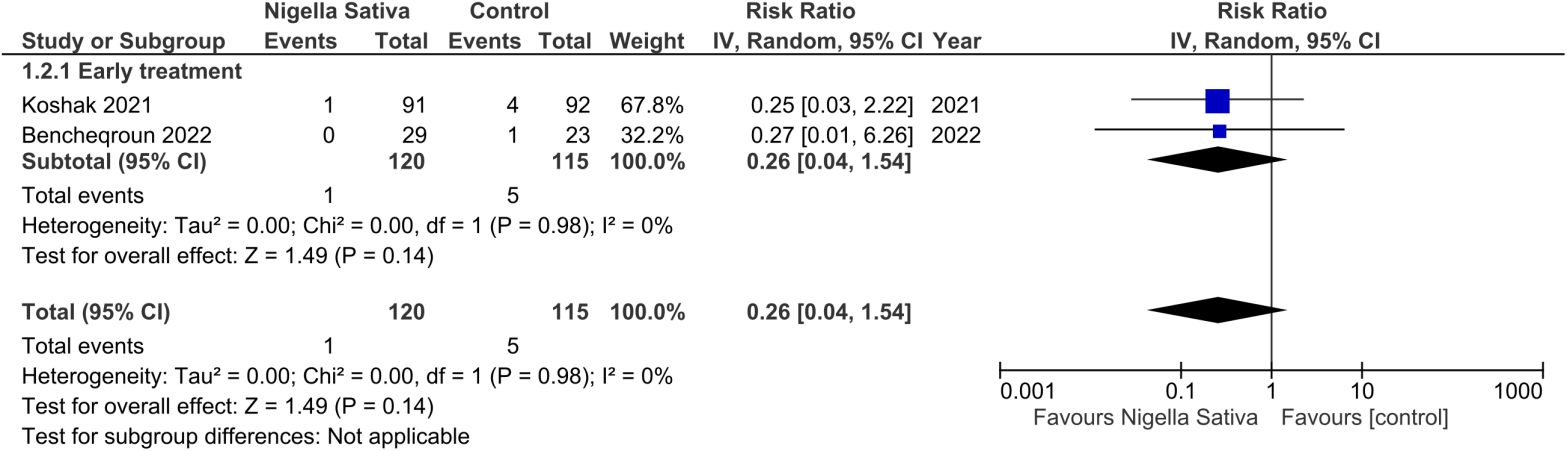
Effect of *Nigella sativa* on the risk of hospitalization in COVID‐19 patients.

There was no significant difference in the rate of hospitalization between the two groups (RR 0.26, 95% CI: 0.04 to 1.54; *I* ^2^ = 0%; Figure 3).

#### 3.3.2 Secondary outcomes

A significant decrease in the rate of viral PCR positivity was observed in the *N. sativa* group (RR 0.62, 95% CI: 0.39 to 0.97; *I* ^2^ = 0%; Figure S2) as compared to the control group. There was no significant difference in the rate of no recovery between the two groups (OR 0.48, 95% CI: 0.20 to 1.15; *I* ^2^ = 84%; Figure S3).

## 4 DISCUSSION

To the best of our knowledge, this is the first comprehensive meta‐analysis to review the effects of *N. sativa* on clinical outcomes in COVID‐19 patients. Our study, in which data from seven RCTs were compiled, demonstrates that *N. sativa* can considerably lower the risk of mortality and increase the rate of virological clearance. However, it did not have a significant effect on the risk of hospitalization and recovery rates.

Our results showing a mortality benefit with the use of *N. sativa* are consistent with the findings of prior studies (Kow et al., 2023). Our study extends and improves their results, first, by incorporating the results from the latest RCTs and second, by evaluating other key outcomes in addition to mortality. However, our findings of a large decrease in mortality need to be interpreted with caution as *N. sativa* did not improve other clinical outcomes as might have been expected given the correlation between mortality and other clinical outcomes, and the interdependent nature of different clinical outcomes. This suggests that *N. sativa* may not be a panacea for treating COVID‐19 patients and further exploration is required to ascertain its potential therapeutic benefits as well as potential drawbacks and side effects.

Historically, *Nigella sativa* has been utilized to treat various disorders due to its antiviral, anti‐inflammatory, and its effects on immunomodulation (Islam et al., 2021). An in vitro study determined the cytotoxic and immune potentiating effects of *N. sativa* after an in vitro cytotoxic screening of *N. sativa* seed extract (Swamy & Tan, 2000). Another study by Hajhashemi et al. found *N. sativa* seed oil extract to have analgesic and anti‐inflammatory effects in male Swiss mice and Wister rats (Hajhashemi et al., 2004). Due to particular constituents such as hederin, thymohydroquinone, and thymoquinone, *N. sativa* possesses potential to combat SARS‐CoV‐2. A study by Mani et al. has demonstrated the potential of *N. sativa* and its constituents in molecular docking studies. The constituent compounds of *N. sativa* can effectively bind with angiotensin‐converting enzyme 2, which acts as a port for the virus's entry into human cells. Consequently, this hindrance impedes infection by preventing efficient binding to ACE2 receptors (Jakhmola Mani et al., 2022). Moreover, thymoquinone exhibits the capability to inhibit cytokine synthesis that promotes inflammation (Hosseinzadeh et al., 2017). However, since safety outcomes were not reported in the trials included, further investigations are required for determining the safety profile of *N. sativa* among individuals afflicted with COVID‐19.

Interpretation of results should be undertaken with caution due to several limitations that this study had. First, the small number of RCTs available may have led to our meta‐analysis being underpowered for some outcomes. Second, some RCTs included in our meta‐analysis were methodologically weak and displayed a high risk of bias. Third, various formulations and combinations of *N. sativa* have been used in the included RCTs. This may have contributed to heterogeneity and constrained the results of the study. Finally, the subjects included had varying degrees of COVID‐19 severity, thus impacting the wide implications of our findings. The strengths of our study include the evaluation of multiple outcomes and the inclusion of data from RCTs only. Nonetheless, we believe that our results are best taken as a lead in directing resources for conducting multicenter research on the effect of *N. sativa* to better characterize the role of *N. sativa* in the management of COVID‐19.

In conclusion, our study demonstrates that early initiation of *N. sativa*, which is an easily available herbal medicine, may decrease the risk of mortality and improve virological clearance in patients with COVID‐19. Further large‐scale RCTs are needed to better understand the effects of *N. sativa* in COVID‐19 patients as an antiviral and anti‐inflammatory agent.

## AUTHOR CONTRIBUTIONS

**Mohammad Umer:** Formal analysis (equal); methodology (equal); resources (equal); writing – original draft (equal). **Aiman Naveed:** Conceptualization (equal); data curation (equal); formal analysis (equal); writing – original draft (equal). **Qanita Maryam:** Data curation (equal); formal analysis (equal); writing – original draft (equal). **Huzaifa Ahmad Cheema:** Conceptualization (equal); methodology (equal); project administration (equal); resources (equal); writing – original draft (equal); writing – review and editing (equal). **Abia Shahid:** Formal analysis (equal); investigation (equal); writing – original draft (equal). **Alaa Hamza Hermis:** Methodology (equal); visualization (equal); writing – review and editing (equal). **Sampath Chinnam:** Resources (equal); writing – review and editing (equal). **Sarya Swed:** Resources (equal); writing – review and editing (equal). **Syeda Sahra:** Supervision (equal); visualization (equal); writing – review and editing (equal).

## FUNDING INFORMATION

No financial support was received for this study.

## CONFLICT OF INTEREST STATEMENT

The authors report no relationships that could be construed as a conflict of interest.

## ETHICS STATEMENT

Research involving human participants and/or animals: No animals or human subjects were used in the current study.

## INFORMED CONSENT

No informed consents were required for the purpose of the current study.

## Supporting information


Figure S1

## Data Availability

The data that support the findings of this study are available on request from the corresponding author. The data are not publicly available due to privacy or ethical restrictions.
