# Supplementary material for: Nigella sativa for the treatment of COVID‐19 patients: A rapid systematic review and meta‐analysis of randomized controlled trials
Source: Food Sci Nutr. 2023 Dec 27;12(3):2061–7. doi: 10.1002/fsn3.3906 (PMC10916652; doi:10.1002/fsn3.3906)

# Supplementary Material

**Nigella sativa for the treatment of COVID-19 patients: a rapid systematic review and meta-analysis of randomized controlled trials**

This supplemental material has been provided by the authors to give readers additional information about their work.

**Figure S1.** Summary of risk of bias of included studies.


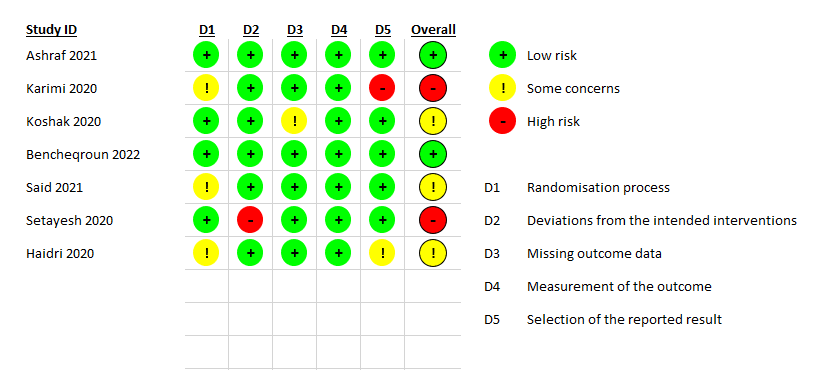


**Figure S2.** Effect of *Nigella sativa* on the rate of viral PCR positivity.


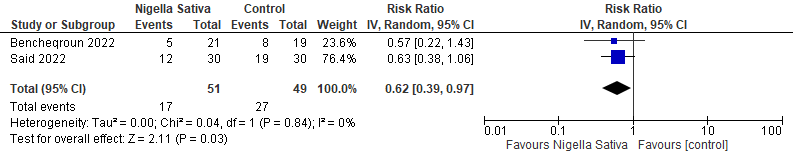


**Figure S3.** Effect of *Nigella sativa* on the rate of no recovery.


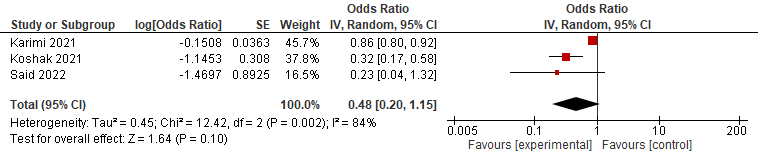

Supplement: Supplementary file 1 — Figure S1 [file FSN3-12-2061-s001.docx]
